# Factors Influencing the Use of Online Symptom Checkers in the United Kingdom: Cross-Sectional Study

**DOI:** 10.2196/65314

**Published:** 2025-09-15

**Authors:** Austen El-Osta, Eva Riboli-Sasco, Mahmoud Al Ammouri, Sami Altalib, Ana Luisa Neves, Azeem Majeed, Benedict Hayhoe

**Affiliations:** 1 Self-Care Academic Research Unit (SCARU), Imperial College London, School of Public Health, 90 Wood Lane, London, W12 0BZ, United Kingdom, 44 2075947604; 2 Department of Public Health and Primary Care, Imperial College London, London, United Kingdom

**Keywords:** digital health, online symptom checker, self-care, symptom checker, United Kingdom, user experience, cross sectional, survey, questionnaire, opinions, perceptions, usability, triage

## Abstract

**Background:**

The National Health Service (NHS) faces increasing strain. Concurrently, demand for health information, consumer empowerment, and health awareness continues to grow. These trends, coupled with the ubiquity of smartphones and internet access, are positioning online symptom checkers (OSCs) as promising tools for preliminary diagnosis and triage. While there is increasing data on the demographics, motivations, and perspectives of current and potential users of OSCs globally, no study has yet quantified or ranked the various factors associated with the use of OSCs in the United Kingdom.

**Objective:**

This study aimed to assess key trends and user perceptions on the usability and effectiveness of OSC in the United Kingdom. We also sought to identify concerns related to the privacy, security, and accuracy of OSCs and to quantify the weight of these various factors on the use of OSCs.

**Methods:**

A cross-sectional survey of UK adults was conducted using an electronic questionnaire. A convenience sample was recruited between February and March 2024 through web-based platforms and personal networks. The survey included questions on awareness, use, perceptions, and concerns regarding OSCs, as well as respondents’ demographics. Responses were pseudo-anonymized and analyzed using univariable and multivariable logistic regression models to assess relationships between demographic factors; perceived usability, reliability, and risks; and OSC use.

**Results:**

The survey collected responses from 634 participants. The majority (543/634, 85.7%) had used OSCs, primarily the NHS 111 service (498/634, 78.6%). Younger age (<46 years old), being female (adjusted odds ratio [aOR] 1.79, 95% CI 1.05‐3.06), and having children (aOR 3.19, 95% CI 1.56‐6.51) were associated with higher odds of using OSCs. Key motivations for using OSCs included understanding symptoms (501/634, 79.0%) and determining the need for medical care (491/634, 77.4%). Key concerns negatively impacting use related to privacy (aOR 0.58, 95% CI 0.35-0.97) and fear of replacing traditional, face-to-face consultations (aOR 0.47, 95% CI 0.26‐0.87). The most important factor found to affect the decision to use OSCs was the perceived ease of use (aOR 8.17, 95% CI 4.25-15.71), followed by the perceived helpfulness in decision-making (aOR 2.96, 95% CI 1.62‐5.42), and respondents’ trust in their diagnostic accuracy (aOR 2.24, 95% CI 1.32‐3.79).

**Conclusions:**

OSCs are widely used in the United Kingdom, particularly the NHS 111 service, driven primarily by ease of use and perceived helpfulness in decision support. However, privacy and security concerns, as well as fears of OSCs replacing traditional consultations, pose significant barriers. Addressing these concerns is crucial for enhancing user trust and maximizing the benefits of OSCs in supporting self-care and improving health care efficiency.

## Introduction

### Overview

The global shortage of health care workers has exacerbated the challenges faced by health systems worldwide. The World Health Organization projects a deficit of 10 million health care workers by 2030. While low- and lower-middle-income countries will be the most affected, all countries will face significant challenges [1]. In the United Kingdom, the National Health Service (NHS) is already under considerable strain [2], which significantly impacts the quality of care and health outcomes for patients [3].

At the same time, there is a growing demand for health information and increasing consumer empowerment [4]. In this context, the reliance on digital health tools has surged, particularly decision support tools including online symptom checkers (OSCs) [5,6]. The ubiquitous access to the internet supports this trend, with 96% of households in the United Kingdom having internet access in 2020 [7], which accelerated following the advent of the COVID-19 pandemic, given the need for health services to avoid face-to-face contact and preserve urgent care capacity [8].

Symptom checkers are available as websites or applications and can generate a prioritized list of potential diagnoses based on the entered symptoms and suggest suitable actions, such as self-care, consulting a general practitioner (GP), or seeking urgent medical care [9]. By providing preliminary diagnostic guidance and triage recommendations, these tools could potentially alleviate some of the burdens on health care systems with the potential to reduce unnecessary health care visits by providing timely medical advice and empowering individuals to make informed decisions about their health [10-12]. Despite this, the diagnostic and triage accuracy varies greatly among OSCs, thus raising concerns and calling for caution [13].

Evidence regarding the sociodemographic profile of OSC users shows that they tend to be young [6,14,15], women [6,14-16], and more educated [6,14,16], whereas confidence in OSC and intentions to discuss recommendations with providers varied by ethnicity [17]. A Chinese study that investigated the use of self-diagnosis health chatbots found a higher prevalence of male users [18], highlighting likely variations across countries. Having a chronic health condition has also been associated with greater use in one study conducted among US users of the Isabel Symptom Checker [16]. A 2022 systematic review on user experiences of symptom checkers identified 8 relevant aspects of user experience that have been explored in the literature, including motivation, trust, acceptability, satisfaction, accuracy, usability, safety/security, and functionality [19]. A recent review identified convenience, supplementing offline medical visits, gaming, and social influence as key motivations for using OSCs, whereas other studies highlighted the quest for a better understanding of the causes of symptoms and support for deciding whether to seek care [16]. Individual factors like internet access, health technology literacy, and awareness of OSC availability also play significant roles in determining user engagement with OSCs [5,12,20], with one UK survey showing higher mean health technology literacy across users of an OSC [21]. Collectively, these findings highlight the importance of enhancing the accuracy, accessibility, usability, and trustworthiness of OSCs to improve user experience and integration into health care services.

While there is a growing body of evidence regarding OSC use, no study has yet quantified or ranked the various factors associated with the use of OSCs in the community setting.

### Objective

This study aimed to identify, characterize, and quantify the factors associated with the use of OSCs among community-dwelling adults in the United Kingdom. Specifically, we sought to (1) assess the demographic characteristics of OSC users, (2) evaluate user perceptions of the usability and effectiveness of OSCs, (3) identify concerns related to the privacy, security, and accuracy of OSCs, and (4) quantify the weight of these various factors on the adoption and use of OSCs.

## Methods

### Ethical Considerations

The Imperial College Research Ethics Committee granted ethical clearance for the study (ICREC# 20IC5974). All experimental protocols were approved by the Imperial College London Research Ethics Committee. All procedures performed in studies involving human participants were in accordance with the ethical standards of the institutional research committee and with the 1964 Helsinki Declaration and its later amendments or comparable ethical standards. Informed consent was obtained from all participants. Consent for publication is not applicable.

All data collected were pseudo-anonymized at the point of submission by assigning unique identifiers, with no directly identifiable personal information (eg, name, address, and IP address) retained in the final dataset. The survey platform (Qualtrics XM) was configured to prevent the collection of IP addresses and location metadata. All data were stored securely on password-protected servers at Imperial College London, accessible only to the study team. Participants were informed that their responses would be confidential, that their participation was voluntary, and that they could withdraw at any time prior to submitting their completed responses. No incentives were offered for participation in the survey unless recruited via the Prolific Academic platform, where participants received a small financial compensation in line with the platform’s fair pay guidelines. This compensation rate was preapproved by the platform and considered appropriate for a brief (under 10-minute) survey. No identifying data were linked to compensation claims.

### Study Design

This cross-sectional study aimed to explore factors influencing the use of OSCs among community-dwelling adults in the United Kingdom. The study used a quantitative methodology using an electronic survey tool (eSurvey).

### Data Collection

This was an open eSurvey, accessible to anyone with the survey link, and required less than 10 minutes to complete. The eSurvey was developed and tested to ensure clarity, usability, and technical functionality before fielding. The link to the eSurvey was active on the Imperial College Qualtrics platform between February 23 and March 25, 2024. Study information was disseminated, including the participant information sheet and link to the survey. Participants were recruited through convenience sampling. The researcher’s personal and professional networks were mobilized to respond and further disseminate the eSurvey among potentially eligible participants. Most participants were recruited via Prolific Academic’s panel [22]. The participant information sheet included information regarding the study’s aims, the protection of participants’ personal data, their right to withdraw from the study at any time, which data were stored, where and for how long, who the investigator was, the purpose of the study, and the survey length.

Participants were informed that this was a voluntary survey. Informed consent was obtained from all participants. Data collected were stored on a secure database at Imperial College London and were only accessible to the research team. All responses were pseudo-anonymized to ensure confidentiality.

The selection of factors that may affect the use of OSCs to be included in the survey was guided by a review of existing literature on the topic. Demographic factors, including age and gender, as well as perceptions such as perceived usability, effectiveness, reliability, accuracy, safety, and privacy, have been identified as influential factors from prior studies [6,16,19,20,23,24].

Before publication, the eSurvey was tested, piloted, and revised internally by the study team. In its final version, the eSurvey comprised a total of 25 questions displayed across 4 screens and gathered data regarding respondents’ awareness, use, and perspectives regarding OSCs, as well as basic demographic information (Textbox 1).

Textbox 1. **Independent and dependent variables.**

**Independent variables**

- Age
- Gender
- Ethnicity
- Highest level of education
- Having children (less than 16 years of age)
- Disability
- Long-term health conditions (LTCs)
- I find symptom checkers easy to use
- Help me make better choices when seeking medical care
- Help me improve my health literacy and support my self-care journey
- Useful when I have limited access to a health care professional (eg, rural setting and out of hours)
- I trust their suggested diagnosis to be accurate
- I trust their triage recommendation (when and where to seek appropriate care) to be accurate
- My general practitioner would encourage me to use symptom checkers
- My family and friends would encourage me to use symptom checkers
- I find using a symptom checker reassuring, and it makes me feel less anxious about my health
- Not yet safe enough to rely solely on them, and may put my health at risk
- I am concerned that using the symptom checker may put my privacy and health information at risk
- I think symptom checkers may increase inequalities between patients
- I am worried about symptom checkers replacing face-to-face or phone consultations

**Dependent variable**

- Using the online symptom checker

The questionnaire underwent preliminary testing with a small group of potential respondents to refine the items based on feedback on clarity and relevance. This included 4 departmental colleagues (2 primary care physicians, a Master of Public Health student, and a researcher), and 8 members of a lay reference group who are diverse in age, gender, and ethnicity. This pilot testing helped in adjusting the survey to better address the study objectives. The study eSurvey was reviewed during beta testing to improve usability and wording of questions, although no attempt was made to validate the tool, given the nature and scope of this study. The final version of the survey instrument is available as Multimedia Appendix 1.

### Data Analysis

Only questionnaires fully completed were included in the analysis. Duplicate entries from the same IP address within a 24-hour period were also eliminated before analysis. Participant characteristics and responses were summarized using total (n) and relative (%) frequencies. For inferential analyses, “strongly agree” and “somewhat agree” were categorized into “agree,” and “strongly disagree” and “somewhat disagree” were categorized into “disagree,” to create binary variables for logistic regression analysis.

In the statistical analysis, we used a series of logistic regression models to examine the associations between various factors and the use of OSC. We conducted both unadjusted and adjusted analyses for four distinct categories of predictors: (1) demographic factors, (2) usability and effectiveness, (3) reliability and accuracy, and (4) risks and concerns. For each category, we first performed univariable logistic regression to assess the unadjusted relationship between the predictors and OSC. Subsequently, we constructed multivariable logistic regression models to account for potential confounding factors. In the adjusted models, we included the following covariates: age, gender, ethnicity, highest level of education, parenting status (having children), disability, and long-term health conditions. For the demographic factors category, we examined the individual associations of each demographic variable with OSC use in the univariable model. In the multivariable model for this category, we included all demographic variables simultaneously to assess their independent effects while controlling for one another. For the other 3 categories (usability and effectiveness, reliability and accuracy, and risks and concerns), the adjusted models included the respective predictors of interest along with the demographic covariates. This approach allowed us to evaluate the relationships between each category of predictors and OSC use, both in isolation and while accounting for potential confounding effects of demographic characteristics.

Results were deemed statistically significant at a *P* value <.05. The odds ratios for these relationships were quantified to understand the influence of each factor on the use of OSCs and compare them. All analyses were performed using STATA (version 17; StataCorp LP). The CHERRIES (Checklist for Reporting Results of Internet E-Surveys) was used to guide reporting [25] (Checklist 1).

## Results

### Participant Characteristics

A total of 641 individuals took part in the survey, with complete responses obtained from 634 respondents. A full description of participants according to age, gender, ethnicity, and educational background is provided in Table 1. The largest proportion of respondents was between 26 and 35 years old (207/634, 32.7%) and female (295/634, 46.5%). The majority identified as White (534/634, 84.2%) and had a college or university degree (453/634, 71.5%). Nearly a third (200/634, 31.5%) had children under 16 years, 15.1% (n=96) reported having a disability, and 22.9% (n=145) had one or more long-term health conditions.

**Table 1.** Respondent characteristics (n=634).

	Values, n (%)
Age (years)	
18‐25	108 (17.0)
26‐35	207 (32.7)
36‐45	156 (24.6)
46‐55	89 (14.0)
56‐65	55 (8.7)
Older than 65	19 (3.0)
Sex	
Male	265 (41.8)
Female	295 (46.5)
Other	65 (10.3)
Prefer not to say	9 (1.4)
Ethnicity	
Asian or Asian British	45 (7.1)
Black, African Caribbean, or Black British	25 (3.9)
White	534 (84.2)
Other ethnic group	25 (3.9)
Prefer not to say	5 (0.8)
Highest level of education	
Primary school or secondary school (up to 16 years)	19 (3.0)
Higher or secondary or further education (A-levels, BTEC, etc)	158 (24.9)
College or university degree	453 (71.5)
Prefer not to say	4 (0.6)
Having children (less than 16 y of age)	
Yes	200 (31.5)
No	434 (68.5)
Disability	
Yes	96 (15.1)
No	509 (80.3)
Prefer not to say	29 (4.6)
Long-term health conditions	
Yes	145 (22.9)
No	453 (71.4)
Prefer not to say	36 (5.7)

### Main Survey Findings

#### Use of OSC

The majority (543/634, 85.7%) of participants had used an OSC, while 14.4% (n=91) reported never having used one. The reasons for nonuse of OSC reported by the largest proportion (41/91, 45.1%) were preference for consulting a health care professional directly rather than using OSC, followed by having never heard of them (36/91, 39.6%); 24.2% (n=22) of respondents did not trust them. However, two-thirds of the nonusers (59/91, 64.8%) expressed a likelihood of using a symptom checker in the future (Table S1 in Multimedia Appendix 2).

The most used OSC was NHS 111, with 78.6% (n=498) of respondents indicating that they had used this tool, followed by Healthline (142/634, 22%). Participants predominantly used symptom checkers before seeking medical advice (533/634, 94.5%), primarily to better understand symptoms (501/634, 79.0%) and to determine the need for medical care (491/634, 77.4%). Most respondents (429/634, 77.9%) indicated that symptom checkers offered recommendations for action or triage. Among those who received such recommendations, a significant proportion (337/429, 79.7%) were directed to seek a consultation with a health care professional. Regarding adherence, most participants (59.8% [n=253]) reported following the recommendations most of the time, 27.7% (n=117) stated they always adhered to the suggestions provided by the symptom checker, while a smaller proportion (52/429, 12.3%) admitted to rarely doing so.

#### Association Between Demographics and Use of OSC

An increase in age was associated with a decrease in OSC use. Specifically, individuals aged 46‐55, 56‐65 and >65 years showed significantly decreased odds of using OSC (adjusted odds ratio [aOR] 0.29, 95% CI 0.11‐0.72), (aOR 0.27, 95% CI 0.10‐0.71), and (aOR 0.22, 95% CI 0.06‐0.78) respectively, compared with the younger [18-25] age group. Similarly, females exhibited higher odds of using OSC compared with males (aOR 1.79, 95% CI 1.05‐3.06). Having children younger than 16 years of age also showed significantly higher odds of using OSC (aOR 3.19, 95% CI 1.56‐6.51) compared with those who do not have children younger than 16 years of age. In contrast, neither ethnicity nor educational background exhibited any statistically significant association with the use of OSC. Similarly, disability and long-term health conditions did not contribute to the outcome of using OSC (Table 2).

**Table 2.** The association between demographics and using an online symptom checker (OSC), adjusted for age, gender, ethnicity, highest level of education, having children, disability, and long-term health conditions.

Variable	Using the OSC			
	Unadjusted		Adjusted	
	OR^a^ (95% CI)	*P* value	OR (95% CI)	*P* value
Age (years)				
18‐25	—^b^	—	—	—
26‐35	1.14 (0.50‐2.59)	.75	1.07 (0.45‐2.54)	.89
36‐45	0.69 (0.31‐1.55)	.37	0.52 (0.21‐1.29)	.16
46‐55	0.35 (0.16‐0.80)	.01	0.29 (0.11‐0.72)	.008
56‐65	0.23 (0.10‐0.54)	.001	0.27 (0.10‐0.71)	.008
Older than 65	0.18 (0.06‐0.55)	.003	0.22 (0.06‐0.78)	.02
Sex				
Male	—	—	—	—
Female	1.98 (1.22‐3.21)	.006	1.79 (1.05‐3.06)	.03
Other	1.28 (0.61‐2.68)	.52	0.81 (0.32‐2.01)	.65
Ethnicity				
White	—	—	—	—
Black, African Caribbean, or Black British	1.97 (0.45‐8.51)	.365	1.16 (0.25‐5.49)	.85
Asian or Asian British	1.75 (0.61‐5.03)	.297	1.41 (0.47‐4.25)	.54
Other ethnic group	0.90 (0.30‐2.69)	.847	0.51 (0.16‐1.63)	.25
Highest level of education				
Primary school or secondary school (up to 16 years)	—	—	—	—
Higher or secondary or further education (A-levels, BTEC, etc)	0.87 (0.24‐3.19)	.83	0.76 (0.19‐3.08)	.70
College or university degree	1.23 (0.35‐4.34)	.75	1.00 (0.26‐3.88)	.99
Having children (less than 16 y of age)				
No	—	—	—	—
Yes	2.62 (1.46‐4.68)	.001	3.19 (1.56‐6.51)	.001
Disability				
No	—	—	—	—
Yes	1.21 (0.63‐2.32)	.57	1.17 (0.50‐2.77)	.72
Long-term health conditions (LTCs)				
No	—	—	—	—
Yes	1.53 (0.85‐2.77)	.16	1.91 (0.94‐3.88)	.07

a OR: odds ratio.

b Reference.

#### Associations Between OSC Use and Users’ Perceptions of OSCs

The main survey findings are shown in Table S1 in Multimedia Appendix 2. The segment below highlights key associations between perceived usability and effectiveness, reliability and accuracy, and risks and concerns with using OSCs.

##### Perceived Usability, Usefulness, and Use of OSCs

Most of the participants found OSCs easy to use (566/634, 89.3%), believed they could help with medical decisions (545/634, 86.0%), and support their health literacy and self-care (537/634, 84.7%). A great proportion (581/634, 94.6%) also agreed that OSCs are useful tools in scenarios with limited access to health care professionals, such as rural settings or out-of-hours situations.

Participants who found the symptom checkers easy to use were more likely to use them compared with those who did not (aOR 8.17, 95% CI 4.25‐15.71). Similarly, individuals who found these tools helpful in making better medical care choices were more likely to use them than those who did not (aOR 2.96, 95% CI 1.62‐5.42). Moreover, those who agreed that the symptom checkers improved health literacy and supported self-care showed a heightened likelihood of use (aOR 2.36, 95% CI 1.30‐4.28). Participants who perceived the OSC as useful in scenarios with limited access to health care professionals were twice as likely to use them compared with those who disagreed (aOR 2.15, 95% CI 1.01‐4.59) (Table 3).

**Table 3.** The association between usability and effectiveness and the use of an online symptom checker (OSC), adjusted for age, gender, ethnicity, highest level of education, having children, disability, and long-term health conditions.

	Using the OSC			
	Unadjusted		Adjusted	
	OR^a^ (95% CI)	*P* value	OR (95% CI)	*P* value
I find symptom checkers easy to use				
Disagree	—^b^	—	—	—
Agree	8.93 (5.16‐15.46)	<.001	8.17 (4.25‐15.71)	<.001
Help me make better choices when seeking medical care				
Disagree	—	—	—	—
Agree	3.05 (1.80‐5.15)	<.001	2.96 (1.62‐5.42)	<.001
Help me improve my health literacy and support my self-care journey				
Disagree	—	—	—	—
Agree	2.66 (1.58‐4.47)	<.001	2.36 (1.30‐4.28)	.005
Useful when I have limited access to a health care professional (eg, rural setting, out of hours)				
Disagree	—	—	—	—
Agree	2.35 (1.22‐4.53)	.011	2.15 (1.01‐4.59)	.05

a OR: odds ratio.

b Reference.

##### Perceived Reliability, Accuracy, and Use of OSCs

Just over half of the respondents expressed confidence and trust in OSCs’ information (363/634, 57.3%). The perceived accuracy of OSCs’ diagnosis was slightly higher (399/634, 63.0%), while 79.2% (n=502) of respondents said they trusted the triage provided by the tools. A total of 69.2% (n=438) of respondents found using a symptom checker reassuring and made them feel less anxious about their health.

Participants who trusted the suggested diagnosis to be accurate were more likely to use the symptom checkers compared with those who disagreed (aOR 2.24, 95% CI 1.32‐3.79). Similarly, individuals who trusted the triage recommendation provided by these tools showed a heightened likelihood of use (aOR 2.33, 95% CI 1.33‐4.06). Participants who found using symptom checkers reassuring and anxiety-reducing were significantly more likely to use them (aOR 3.85, 95% CI 2.28‐6.50). Additionally, the encouragement from family and friends to use symptom checkers significantly influenced their use (aOR 2.05, 95% CI 1.24‐3.41). However, encouragement from GPs was not significantly associated with the use of symptom checkers (aOR 1.34, 95% CI 0.79‐2.24) (Table 4).

**Table 4.** The association between reliability and accuracy and the use of an online symptom checker (OSC), adjusted for age, gender, ethnicity, highest level of education, having children, disability, and long-term health condition.

	Using the OSC			
	Unadjusted		Adjusted	
	OR^a^ (95% CI)	*P* value	OR (95% CI)	*P* value
I trust their suggested diagnosis to be accurate				
Disagree	—^b^	—	—	—
Agree	2.14 (1.36‐3.34)	.001	2.24 (1.32‐3.79)	.003
I trust their triage recommendation (when and where to seek appropriate care) to be accurate				
Disagree	—	—	—	—
Agree	2.55 (1.58‐4.12)	<.001	2.33 (1.33‐4.06)	.003
My general practitioner (GP) would encourage me to use symptom checkers				
Disagree	—	—	—	—
Agree	1.58 (0.99‐2.52)	.054	1.34 (0.79‐2.24)	.28
My family and friends would encourage me to use symptom checkers				
Disagree	—	—	—	—
Agree	2.08 (1.33‐3.26)	.001	2.05 (1.24‐3.41)	.005
I find using a symptom checker reassuring, and it makes me feel less anxious about my health				
Disagree	—	—	—	—
Agree	3.35 (2.12‐5.27)	<.001	3.85 (2.28‐6.50)	<.001

a OR: odds ratio.

b Reference.

##### Perceived Risks and Concerns and Use of OSCs

Reported concerns included safety (481/634, 75.9%), privacy (258/634, 41.7%), and exacerbating inequalities (257/634, 41.6%). More than half of the respondents (417/634, 65.9%) worried about replacing traditional consultations, and more than a quarter (170/634, 26.9%) would not feel confident discussing the outcomes of their symptom checker consultation with their GP. Participants who agreed that symptom checkers are not yet safe enough to rely solely on them and may put their health at risk did not show a significant association with use compared with those who disagreed (aOR 0.59, 95% CI 0.30‐1.17). However, concerns regarding privacy and health information security were significantly inversely associated with use, with individuals expressing such worries being less likely to use symptom checkers (aOR 0.58, 95% CI 0.35‐0.97). Similarly, those who believed that symptom checkers might increase inequalities between patients were less likely to use them (aOR 0.47, 95% CI 0.28‐0.79). Concern about OSCs replacing face-to-face or phone consultations was significantly associated with decreased use (aOR 0.47, 95% CI 0.26‐0.87) (Table 5).

**Figure 1.**
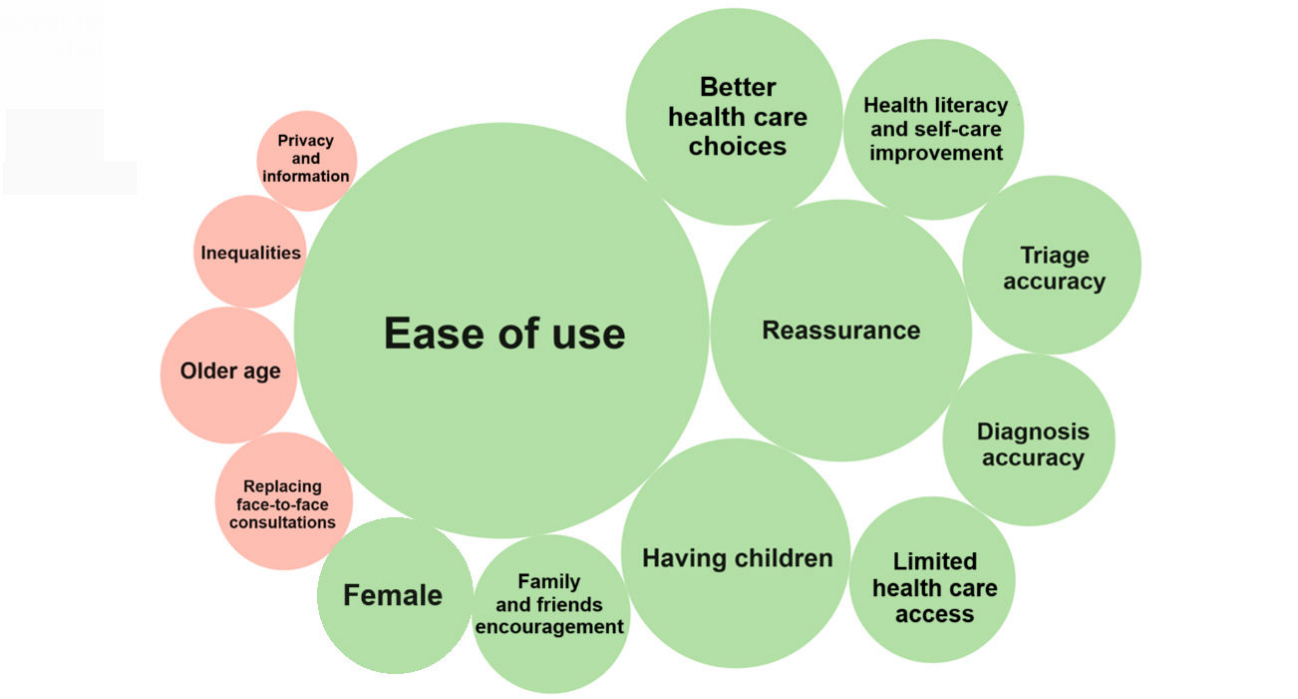
Factors that significantly influence the use of an online symptom checker. The size of the bubbles is proportional to the odds ratios of each factor’s influence, where green depicts factors that increase the likelihood, and red indicates factors that decrease the likelihood of using online symptom checkers.

Figure 1 highlights the relative weight of each factor on the use of OSCs. The main factor that significantly increased the probability of using OSCs was the tools’ ease of use (aOR 8.17, 95% CI 4.25‐15.71). This was followed by feeling reassured by using the tool and having children. Users of these decision support tools also usually thought of OSCs as helpful in influencing and improving their health care choices, their health literacy, and self-care capacity. Encouragement from friends and family, limited health care access, being female, and trust in the triage and diagnostic accuracy of OSCs were also important factors.

Demographic factors associated with decreased odds of using OSCs included male gender and older age. Concerns regarding privacy and data security, as well as the risk of increased inequalities and loss of face-to-face consultations due to OSCs, were also identified as reducing the likelihood of using these tools (Figure 1).

**Table 5.** The association between risks and concerns and the use of an online symptom checker (OSC), adjusted for age, gender, ethnicity, highest level of education, having children, disability, and long-term health conditions.

	Using the OSC			
	Unadjusted		Adjusted	
	OR^a^ (95% CI)	*P* value	OR (95% CI)	*P* value
Not yet safe enough to rely solely on them, and may put my health at risk				
Disagree	—^b^	—	—	—
Agree	0.69 (0.39‐1.21)	.19	0.59 (0.30‐1.17)	.13
I am concerned that using the symptom checker may put my privacy and health information at risk				
Disagree	—	—	—	—
Agree	0.46 (0.29‐0.71)	.001	0.58 (0.35‐0.97)	.04
I think symptom checkers may increase inequalities between patients				
Disagree	—	—	—	—
Agree	0.59 (0.38‐0.92)	.02	0.47 (0.28‐0.79)	.005
I am worried about symptom checkers replacing face-to-face or phone consultations				
Disagree	—	—	—	—
Agree	0.50 (0.29‐0.84)	.010	0.47 (0.26‐0.87)	.015

a OR: odds ratio.

b Reference.

## Discussion

### Summary of Principal Findings

This study investigated the factors that influence the use of OSC through a cross-sectional survey of community-dwelling adults in the United Kingdom. Most participants (545/634, 86%) had used a symptom checker at some point, with the NHS 111 platform being the most widely used (501/634, 79%), followed by Healthline (142/634, 22%). NHS 111 is a free-to-use service provided by the UK’s NHS that offers both online and telephone triage for urgent but nonemergency health concerns. This platform guides users through a series of questions about their symptoms, providing advice on the next steps to take, which may include self-care, contacting a GP, or seeking emergency services. The service aims to reduce unnecessary visits to emergency departments while ensuring patients receive appropriate care [26]. Healthline, on the other hand, is a US-based health information website that provides comprehensive, evidence-based health content covering various topics, conditions, and wellness information [27]. These tools and other OSCs named by respondents to our survey were predominantly used before seeking medical advice (602/634, 95%), primarily to better understand symptoms and to determine the need for care.

We found that while most OSC nonusers expressed a strong likelihood of future use, respondents had varying concerns regarding the accuracy of information, safety, privacy, and the potential increase of inequalities. Older individuals (aged between 46‐55, 56‐65, and older than 65 years) showed significantly decreased odds of using OSC compared with the 18‐25 years age group, independently of other variables. However, females exhibited higher odds of using these tools compared with males, and individuals with children younger than 16 years of age were more likely to use OSC compared with those who did not have children. Further, although trust in the accuracy of diagnoses and triage recommendations, as well as encouragement from family and friends, positively influenced use, concerns regarding privacy, health information security, inequalities, and the potential displacement of traditional consultations were significantly associated with decreased use (Figure 1). No significant associations were found between the use of OSCs and encouragement from GPs to use these tools, concerns about the safety of relying solely on symptom checkers, or potential health risks. These findings highlight the complex nature of the many factors that could influence the use of OSCs in the contemporary setting.

### Study Strengths and Limitations

A key strength of this study is that it examined a wide range of variables, including demographics, motivations, perceived effectiveness, reliability, and concerns regarding OSC and associations with their use, providing valuable insights into the complexities surrounding the wide-scale adoption and diffusion of these tools in the contemporary setting. While supporting existing evidence on this topic, we identified additional factors associated with the use of OSCs, including the perceived family and friends support, as well as having children. By using regression models, we were also able to quantify the associations between the use of OSCs and a variety of factors, including both demographic factors and respondents’ perceptions regarding the usability, safety, accuracy, and concerns associated with OSCs. Crucially, we identified significant predictors of use while controlling for potential confounders and enhancing the internal validity. The study’s relatively large and diverse sample also closely reflects the ethnic distribution of the UK population in terms of broad categories [28] as well as the prevalence of long-term conditions and disabilities.

The principal limitation of this study is its cross-sectional nature, as it cannot establish causality and temporal relationships between the factors examined. The other main limitation is concerned with the sampling method. While convenience sampling allowed for efficient data collection and provided initial insights into the perceptions of OSC users, it potentially limits the generalizability of our findings. Future studies should consider using probability sampling techniques to ensure a more representative sample of the general population, thereby enhancing the external validity of the results. Another key limitation is that the study used a single-item measure to assess the usability of OSC. Recognizing the complexity of usability as a construct, we acknowledge that this simplified approach may not capture all dimensions of usability effectively, but we deemed it sufficient for the purpose of keeping the number of questions in the survey to a minimum, especially as we sought to measure responses to other factors. Future research would benefit from incorporating multidimensional, validated usability scales to provide a more detailed understanding of user interactions with digital health tools.

As the survey was only accessible via web, it is likely that potentially eligible participants with limited access to the internet or those less confident with digital technology may have been excluded and their views absent or underreported. In addition, we used a convenience sample and could not measure response rates. The voluntary nature of the survey meant that respondents with an interest in or previous experience with OSCs might have been more likely to take part, which could explain the high prevalence of people who had previously used an OSC (543/634, 85.6%). While we adjusted for several demographic factors in our analyses, the possibility of unmeasured confounding cannot be ruled out, which may influence the observed associations. Our primary analysis also did not extensively explore interactions between covariates, potentially overlooking complex relationships between factors associated with OSC use. We acknowledge also that, as this study relied on self-reported data, this may be subject to recall and social desirability biases, and for this reason, participants’ responses regarding their use patterns, preferences, and adherence to recommendations may therefore not fully reflect their actual behaviors. Because these limitations are common in survey studies, longitudinal studies are indicated to follow up users over a longer time horizon to better understand how their interactions with OSCs evolve and how these tools may influence their health behaviors, lifestyle choices, health care use, and health outcomes.

### Comparison With Existing Literature

The findings of this study are in line with prior research, including primary studies and reviews reporting on the sociodemographics of OSC users who tend to be young [6,12,14,15], women [6,12,14-16], and with higher education levels [6,12,14,16], and higher digital literacy scores [21]. Although having a chronic health condition or a disability was associated with greater use in a study by Meyer et al [16], we did not find this association in this study’s sample. Regarding the motivations for using OSCs, a better understanding of the causes of symptoms has also been found to be the primary motivation among US users of the Isabel Symptom Checker [16], followed by support for deciding whether to seek care.

The finding that users of OSCs tend to find these tools easy to use and helpful was corroborated by Meyer et al [16] and Pairon et al [6]. The strong correlation identified in this study highlights the importance of user-friendly interfaces in promoting the adoption of OSCs. The review by Pairon et al [6] also emphasized that users value OSCs for their ability to support health-related decisions, especially in determining whether to seek medical care. The results of this study reinforce this, as individuals who perceived OSCs as helpful in making better medical care choices were nearly 3 times more likely to use them.

Compliance with OSC recommendations has also been a point of focus in the literature. Previous studies reported varying levels of adherence to OSC advice, with compliance rates ranging from 57% to 67% [6], although in our study, we report a significantly higher compliance rate, with 87.9% (557/634) of participants following OSC recommendations most or all of the time. This higher rate of adherence may reflect an increasing reliance on digital health tools, particularly in the context of the COVID-19 pandemic, which has accelerated the adoption of telemedicine and web-based health resources. Further research is needed to continue monitoring these trends and to explore the long-term impact of OSC use on health care outcomes.

Finally, issues relating to perceived accessibility, accuracy, security, and privacy of OSCs were also identified by Aboueid et al [20] in their qualitative study exploring young adults’ perspectives on the use of OSCs. Most of their respondents thought of OSCs as more useful for self-triage than self-diagnosis, which reflects the fact that only 62.9% (399/634) of the respondents in this study trusted the diagnosis provided by the OSC, compared with 78.9% (500/634) for the triage suggestion.

### Implications for Research

Although this study identified demographic disparities in the use of OSCs, further research is warranted to understand the underlying reasons for these disparities. Research focusing on the sociocultural factors, digital literacy, and health care-seeking behaviors among different demographic groups could provide valuable insights into addressing disparities and promoting equitable access to OSC. In addition, this study’s findings highlight the importance of usability, effectiveness, and trust in driving the adoption and use of OSCs. Future research could investigate the specific features and functionalities of these tools, such as user interface design and decision support algorithms that contribute to their perceived usability, effectiveness, and adherence to the recommendations.

Our study highlighted concerns regarding the privacy and health information security of OSC, which could impact their acceptance and use, necessitating the development of robust frameworks, regulatory standards, and guidelines for OSC platforms to ensure transparency, accuracy, and user privacy. Additionally, studies investigating the potential implications of OSC on health care inequalities and the doctor-patient relationship are essential for informing policy and practice, whereas research exploring effective strategies for educating users about the capabilities and limitations of these tools, as well as enhancing communication and collaboration between users and health care providers, could help build trust and confidence in OSC.

Future research should focus on understanding the sociocultural factors influencing OSC use and developing strategies to address privacy and security concerns. Additionally, efforts to improve the usability and reliability of OSCs, alongside targeted interventions to promote equitable access, are essential for integrating these tools effectively into the health care system. By addressing these issues, OSCs can play a key role in supporting self-care and improving health care accessibility and efficiency in the United Kingdom.

### Conclusions

This study provides insights into the factors influencing the use of OSCs in the United Kingdom, highlighting both their increasing and widespread adoption and some of the concerns associated with these digital health tools that will likely be crucial to inform the self-care journey of people in different settings and from different walks of life. While most of the respondents to our survey of British adults used OSCs, particularly the NHS 111 service, primarily for understanding symptoms and determining the need for medical care, younger individuals, females, and those with children are more likely to use OSCs overall. Ease of use, perceived helpfulness in medical decision-making, and trust in the accuracy of diagnoses and triage recommendations are key factors driving OSC use, but these are coupled to concerns about privacy, health information security, and the potential for OSCs to exacerbate health care inequalities, posing significant barriers to their adoption. The fear of OSCs replacing traditional consultations with health care professionals remains common among users, and these concerns must be addressed to enhance user trust and maximize the benefits of OSCs in health care delivery.

## Supplementary material

10.2196/65314 Multimedia Appendix 1 Survey export (raw xls).

10.2196/65314 Multimedia Appendix 2 Main survey findings.

10.2196/65314 Checklist 1 CHERRIES checklist.

## Abbreviations

aOR: adjusted odds ratio
CHERRIES: Checklist for Reporting Results of Internet E-Surveys
GP: general practitioner
NHS: National Health Service
OSC: online symptom checker
