# Supplementary material for: Factors Influencing the Use of Online Symptom Checkers in the United Kingdom: Cross-Sectional Study
Source: JMIR Form Res. 2025 Sep 15;9:e65314. doi: 10.2196/65314 (PMC12435756; doi:10.2196/65314)
Supplement: Multimedia Appendix 2 [file formative-v9-e65314-s002.pdf]

Table S1: Main survey findings

	N	(%)
**Have you used an OSC?**		
Yes	543	85.65
No	91	14.35
**Which of the following OSC (OSC) have you already used? (n=634) ***		
Ada	10	1.58
Babylon / eMed	29	4.57
Family Doctor	42	6.62
Healthline	142	22.40
Isabel	3	0.47
NHS 111	498	78.55
Patient.info	69	10.88
Sharecare	1	0.16
Symptomate	5	0.79
Your.MD / Healthily	95	14.98
Other	27	4.26
**Why have you never used a symptom checker (n=91)?**		
I had never heard of them	36	39.56
I do not trust them	22	24.18
I don't understand how they work	11	12.09
I have limited access to internet and/or digital devices	0	0
I prefer consulting a healthcare professional directly	41	45.05
Other	7	7.69
**If you had access to one, how likely would you use a symptom checker in the future? (n=91)**		
Very unlikely	7	7.69
Unlikely	25	27.47
Likely	55	60.44
Very likely	4	4.40
**How likely would you be to use an OSC for a child you care for (e.g.: your child, grandchild, niece/nephew, babysitting...)? (n=634)**		
Very unlikely	26	4.10
Unlikely	54	8.52
Likely	251	39.59
Very likely	254	40.06
Not applicable	49	7.73
**If you were to use a symptom checker, how would you prefer to access it? (n=634)**		
Website	213	33.60
Phone app/ tablet	131	20.66
Both	290	45.74
**When would you typically use a symptom checker? (n=634)***		
Instead of seeing a doctor	154	24.29
Before seeing a doctor	599	94.48
After seeing a doctor	51	8.04
None of the above	9	1.42
**For what reason would you use a symptom checker? (n=634)***		
To help me determine if I have a medical emergency & should seek immediate medical care	430	67.82
To help me determine whether or not I need to book an appointment with a healthcare professional (doctor, nurse, specialist, etc)	491	77.44
To better understand what could be causing my symptoms	501	79.02
To get medical advice without going to the doctor	274	43.22
To confirm the diagnosis made by my doctor	86	13.56
Other	6	0.95
None of the above / I would never use a symptom checker	3	0.47
**Reflecting on your past experience, did the symptom checker**		

**recommend action or triage option (e.g., see doctor, or seek urgent care)? (n=543)**		
Yes	423	77.90
No	120	22.10
**Which of the following recommendation(s) have you already received from a symptom checker? (n=423) ***		
Self-care (including taking over-the-counter medication, rest, etc.)	254	60.05
See a pharmacist or nurse	157	37.12
See GP / doctor	337	79.66
Seek urgent care (e.g., go to A&E)	152	35.93
Call an ambulance	32	7.57
Other	3	0.71
**Have you followed the recommendation(s)? (n=423)**		
Never	1	0.24
Rarely	52	12.29
Most of the time	253	59.81
Always	117	27.66
**Who do you think is more likely to make a mistake about your condition or triage? (n=634)**		
A healthcare professional	16	2.52
A symptom checker	393	61.99
Both equally	139	21.92
I am not sure	86	13.56
**Do you think GPs should ask patients to use a symptom checker before booking an appointment? (n=634)**		
Yes	203	32.02
No	431	67.98
**To what extent do you agree with the following statements regarding the usability & effectiveness of symptom checkers? (n=634)**		
**I find symptom checkers easy to use**		
Strongly agree	208	32.81
Somewhat agree	358	56.47
Somewhat disagree	59	9.31
Strongly disagree	9	1.42
**I think anyone has the ability to use a symptom checker**		
Strongly agree	132	20.82
Somewhat agree	278	43.85
Somewhat disagree	174	27.44
Strongly disagree	50	7.89
**Using symptom checkers can help me make better choices when seeking medical care**		
Strongly agree	126	19.87
Somewhat agree	419	66.09
Somewhat disagree	75	11.83
Strongly disagree	14	2.21
**Using symptom checkers can help me improve my health literacy & support my self-care journey**		
Strongly agree	132	20.82
Somewhat agree	405	63.88
Somewhat disagree	84	13.25
Strongly disagree	13	2.05
**Symptom checkers give me extra information I can't find elsewhere**		
Strongly agree	96	15.14
Somewhat agree	286	45.11
Somewhat disagree	203	32.02
Strongly disagree	49	7.73

**Symptom checkers are useful when I have limited access to a healthcare professional (e.g., rural setting, out of hours)**		
Strongly agree	270	42.59
Somewhat agree	311	49.05
Somewhat disagree	37	5.84
Strongly disagree	16	2.52
**Since COVID-19, I would rather check my symptoms before unnecessarily going to see a healthcare professional**		
Strongly agree	164	25.87
Somewhat agree	277	43.69
Somewhat disagree	147	23.19
Strongly disagree	46	7.26
**To what extent do you agree with the following statements regarding the reliability & accuracy of symptom checkers? (n=634)**		
**I trust their suggested diagnosis to be accurate**		
Strongly agree	23	3.63
Somewhat agree	383	60.41
Somewhat disagree	198	31.23
Strongly disagree	30	4.73
**I trust their triage recommendation (when & where to seek appropriate care) to be accurate**		
Strongly agree	74	11.67
Somewhat agree	428	67.51
Somewhat disagree	113	17.82
Strongly disagree	19	3.00
**I am prepared to follow their recommendations without checking with my doctor**		
Strongly agree	40	6.31
Somewhat agree	256	40.38
Somewhat disagree	259	40.85
Strongly disagree	79	12.46
**My GP would encourage me to use symptom checkers**		
Strongly agree	44	6.94
Somewhat agree	231	36.44
Somewhat disagree	299	47.16
Strongly disagree	60	9.46
**My family and friends would encourage me to use symptom checkers**		
Strongly agree	43	6.78
Somewhat agree	327	51.58
Somewhat disagree	209	32.97
Strongly disagree	55	8.68
**I find using a symptom checker reassuring & it makes me feel less anxious about my health**		
Strongly agree	73	11.51
Somewhat agree	366	57.73
Somewhat disagree	151	23.82
Strongly disagree	44	6.94
**A phone call with a nurse will be more accurate & useful than using a symptom checker**		
Strongly agree	147	23.19
Somewhat agree	317	50.00
Somewhat disagree	157	24.76
Strongly disagree	13	2.05
**To what extent do you agree with the following statements regarding the risks & concerns associated to symptom checkers? (n=634)**		

**Symptom checkers are not yet safe enough to rely solely on them & may put my health at risk**		
Strongly agree	202	31.86
Somewhat agree	279	44.01
Somewhat disagree	145	22.87
Strongly disagree	8	1.26
**I am concerned using the symptom checker may put my privacy & health information at risk**		
Strongly agree	66	10.41
Somewhat agree	192	30.28
Somewhat disagree	291	45.90
Strongly disagree	85	13.41
**I think symptom checkers may increase inequalities between patients**		
Strongly agree	61	9.62
Somewhat agree	203	32.02
Somewhat disagree	304	47.95
Strongly disagree	66	10.41
**I am worried about symptom checkers replacing face-to-face or phone consultations**		
Strongly agree	182	28.71
Somewhat agree	236	37.22
Somewhat disagree	165	26.03
Strongly disagree	51	8.04
**I would not feel confident discussing the outcomes of my symptom checker consultation with my GP**		
Strongly agree	39	6.15
Somewhat agree	132	20.82
Somewhat disagree	315	49.68
Strongly disagree	148	23.34

*= Multiple choice question (any unit of interest is the number of answers and not the number of respondents)
